# Ambient fine particulate matter of diameter ≤ 2.5 μm and risk of hemorrhagic stroke: a systemic review and meta-analysis of cohort studies

**DOI:** 10.1007/s11356-021-13074-7

**Published:** 2021-03-10

**Authors:** Kai Zhao, Jing Li, Chaonan Du, Qiang Zhang, Yu Guo, Mingfei Yang

**Affiliations:** 1 Graduate School, Qinghai University, Xining, 810016 Qinghai China; 2 Department of Community Health Education, Institute for Health Education of Qinghai Province, Xining, 810000 Qinghai China; 3 Qinghai Provincial People’s Hospital, Qinghai, 810007 China

**Keywords:** Hemorrhagic stroke, PM_2.5_, Hazard ratio, Meta-analysis

## Abstract

Ambient fine particulate matter of 2.5 μm or less in diameter (PM_2.5_) of environment contamination is deemed as a risk factor of cerebrovascular diseases. Yet there is still no explicit evidence strongly supporting that PM_2.5_ with per unit increment can increase the risk of hemorrhagic stroke (HS). Literatures were searched from PubMed, Cochrane, and Embase. After the systemic review of relevant studies, random effects model was used to perform meta-analysis and to evaluate the association between PM_2.5_ and risk of HS. Seven cohort studies were finally included, involving more than 6 million people and 37,667 endpoint events (incidence or mortality of HS). Total scores of quality assessment were 50. Pooled hazard ratio (HR) for crude HRs was 1.13 (95%CI: 1.09–1.17) (CI for confidence interval). Pooled HR of subgroup analysis for current smoking with exposure to growing PM_2.5_ was 1.14 (95%CI: 0.92–2.15) and for never and former smoking was 1.04 (95%CI: 0.74–1.46). Ambient PM_2.5_ level is significantly associated with the risk of HS, which might be a potential risk factor of HS. Smoking does not further increase the risk of HS under exposure of PM_2.5_.

## Introduction

Ambient air pollution is a major and significant environmental risk to the health of people in both cities and rural areas (https://www.who.int/). According to the data from the World Health Organization, 58% of premature deaths were related to outdoor air pollution. Especially, the fatal effects of air pollution were presented as ischemic heart diseases and strokes resulted from exposure to fine particulate matter of 2.5 μm or less in diameter (PM_2.5_). Moreover, hemorrhagic stroke (HS) accounted for one-third of strokes. Therefore, PM_2.5_ might be closely related to risk of HS. Recently, Sheng Yuan (Yuan et al. 2019) and other collaborators conducted a meta-analysis and concluded that the long-term exposure to PM_2.5_ was an important risk factor for stroke. However, the relationship between risk of HS and ambient PM_2.5_ exposure has not been accurately confirmed. We supposed that some risk factors of ischemic stroke such as smoking were not associated with risk of HS. Thus, we searched recent cohort studies from open medical database to conduct a meta-analysis and to elucidate the relationship between risk of HS and increase of PM_2.5_.

## Methods

This systemic review was performed according to the protocol published on the database of International Platform of Registered Systematic Review and Meta-analysis Protocols (INPLASY, https://inplasy.com/, registration number: INPLASY202050022, DOI number: 10.37766/inplasy2020.5.0022).

### Literature search

Literature search was conducted from PubMed, Cochrane, and Embase databases. While making the strategy of literature search, publication time, regions, language, and human species were not restricted. The MeSH term was defined as “stroke” combined with “particulate matter.” Moreover, types of literature were not limited. Titles, keywords, abstracts, and relevant information of publication were downloaded to the software Endnote X9 (BId 12062) for article management. All literatures were independently reviewed and analyzed by two authors (Kai Zhao and Yu Guo). If there is inconsistency between them, another author (Mingfei Yang) would be consulted or the literature search strategy would be modified to reach a consensus.

The full strategy of searching literature in PubMed and Cochrane was as follows: (((((((((Stroke[MeSH Terms]) OR Strokes[Title/Abstract]) OR “Cerebrovascular Accident*”[Title/Abstract]) OR CVA*[Title/Abstract]) OR Apoplexy[Title/Abstract]) OR “Vascular Accident*, Brain”[Title/Abstract]) OR “Brain Vascular Accident*”[Title/Abstract]) OR stroke[Title/Abstract])) AND (((((((((((“Air Pollution”[MeSH Terms]) OR “Pollution, Air”[Title/Abstract]) OR “Air Quality”[Title/Abstract]) OR “Particulate Matter”[MeSH Terms]) OR “Airborne Particulate Matter”[Title/Abstract]) OR “Particulate Matter, Airborne”[Title/Abstract]) OR “Air Pollutant*, Particulate”[Title/Abstract]) OR “Particulate Air Pollutant*”[Title/Abstract]) OR “Pollutant*, Particulate Air”[Title/Abstract]) OR “Ambient Particulate Matter”[Title/Abstract]) OR “Particulate Matter, Ambient”[Title/Abstract]).

The full strategy of searching literature in Embase was as follows: (exp Stroke or Stroke$:ab,ti or 'Cerebrovascular Accident$':ab,ti or CVA$:ab,ti or Apoplexy:ab,ti or 'Vascular Accident$, Brain':ab,ti or 'Brain Vascular Accident$':ab,ti or stroke:ab,ti) and (exp 'Air Pollution' or 'Pollution, Air':ab,ti or 'Air Quality':ab,ti or exp 'Particulate Matter' or 'Airborne Particulate Matter':ab,ti or 'Particulate Matter, Airborne':ab,ti or 'Air Pollutant$, Particulate':ab,ti or 'Particulate Air Pollutant$':ab,ti or 'Pollutant$, Particulate Air':ab,ti or 'Ambient Particulate Matter':ab,ti or 'Particulate Matter, Ambient':ab,ti).

### Inclusion and exclusion criteria

Articles from three electronic databases were pooled to complete the preparation of screening. After deleting the duplications, we established two initial criteria for inclusion and exclusion, respectively. Inclusion criteria included cohort studies, “stroke” and “particulate matter” found in title or abstract. Exclusion criteria: studies without cohort design, animals, or special people enrolled as subjective of studies, and no MeSH term in abstract. After completion of initial screening, final criterion was used to select studies for meta-analysis. Final inclusion criterion for full texts of article: “PM_2.5_” and “hemorrhagic stroke.” Final exclusion criterion: absence of hazards ratio (HR) or “increase of PM_2.5_.” Screening of articles was conducted by one author (Kai Zhao) and examined and verified by another author (Jing Li). The third author (Mingfei Yang) would settle disagreement.

### Data extraction

The data of first author’s name, publication year, study region, exposure, period, total numbers of participants, gender ratio (percent of male in all of the participants), definition of endpoints, increase extent of PM_2.5_, and HR with 95% confidence interval (CI) were extracted. When there were different results of HR with 95%CI for different models adjusted for various covariates, data and relative information of all models were extracted.

### Quality assessment

All enrolled studies were independently assessed by two authors (Kai Zhao and Yu Guo) according to Newcastle-Ottawa Quality Assessment Scale Cohort Studies (NOS). All eligible studies were assessed from three main aspects: representativeness of participants included, levels of ambient PM_2.5_, and other factors that could influence the outcome, objectivity, and accuracy of determination of endpoint.

### Data standardization

There were various factors that were adjusted to different proportional hazard models. If the coefficient of each factor was supplied in full text, crude data would be restored by conversion formula. The data adjusted by the least factors would not be selected to perform data processing, unless there was no way to acquire crude data. Likewise, data adjusted by different factors would be processed in the same way.

### Statistical analysis

The pooled HR and 95%CI was calculated by random effects model. I-square (I^2^) was used to test the heterogeneity. Funnel plot asymmetry and Egger’s regression were used for detecting publication bias. Sensitivity analysis was used to attenuate heterogeneity, in which each study was omitted one by one and the pooled HR of the rest studies was retrieved or the random effects model was switched to fixed effects model. Meta-analysis was completed through corresponding modules in Software for Statistics and Data Science (version 15.1; College Station, TX 77845 USA). All *p* values were two-sided with a significant level at 0.05.

## Results

**Fig. 1:**
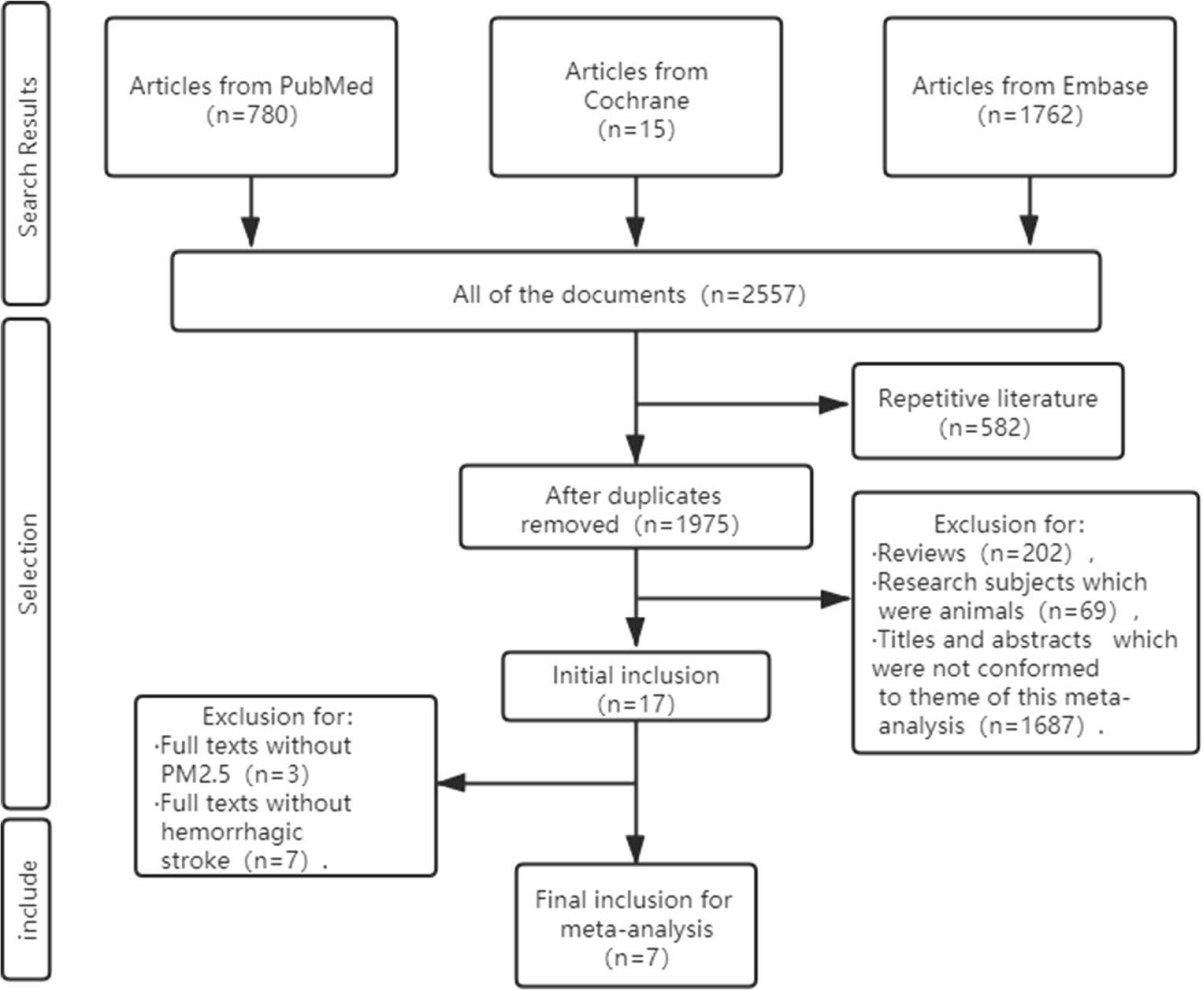
Process of searching for studies and screening

Overall, 2557 publications were obtained from the literature search (780 from PubMed, 15 from Cochrane, and 1762 from Embase). After removing duplicates, 1975 articles were initially included. After excluding reviews, researches of animals, and titles and abstracts that were not conformed to the theme of “PM_2.5_” and “Stroke,” 17 articles were retained for final screening. After excluding full texts without “PM_2.5_” and “Hemorrhagic Stroke” or other types of studies except for cohort studies, ultimately, 7 studies were included in this meta-analysis. Of these 7 studies, 6 studies (Huang et al. 2019; Cai et al. 2018; Downward et al. 2018; Qiu et al. 2017; Shin et al. 2019; Noh et al. 2019) reported the HR between exposure to the circumstance contaminated by growing PM_2.5_ and occurrence of HS or hemorrhagic cerebrovascular accident except that one study (Kim et al. 2018) mentioned the mortality caused by HS and used it as the endpoint. Detailed information of screening is exposed to Fig. 1. The characteristics of eligible studies are shown in Table 1.

**Table 1:** Characters of studies included finally

Authors	Year of publication	Area	Increase of PM_2.5_ (μg/m^3^)	Study population	Exposure period	Age (years)	Gender (male, %)	Endpoints cases	Definition of endpoint
Keyong Huang et al.	2019	China	10	117575	2000–2015	50.9 ± 11.8	41.0	1019	ICD-10 I60,I61,I62
Yutong Cai et al.	2018	UK	1.4	355732	1993–2013	52.9 ± 10.6	42.0	307	ICD-9 431;ICD-10 I60,I61,I62
George S Downward et al.	2018	Netherlands	5	33831	1993–2010	50.0 ± 11.0	23.0	241	ICD
Jong-Hun Kim et al.	2018	Korea	10	40% of national population	1990–2013	Null	Null	12,832	ICD-10 I60,I61,I62,I690,I691, I692,I694
Hong Qiu et al.	2017	Hong Kong, China	10	66820	1998–2001	72	34.1	1175	ICD-9 430,431
Saeha Shin et al.	2019	Canada	4.1	5071956	2001–2015	53.2 ± 12.9	48.0	21,581	ICD-9 430,431;ICD-10 I60,I61
Juhwan Noh et al.	2019	Korea	10	62676	2002–2013	≥ 20	49.3	512	ICD-10 I60–I62

### Quality assessment for included studies

All the studies were assessed by NOS. Six studies were scored 7 points and one study was scored 8 points. All studies were scored in representativeness of the exposed cohort, ascertainment of exposure, comparability of cohorts on the basis of the design or analysis, assessment of outcome, and a long follow-up duration. Only one study (Huang et al. 2019) was scored in adequacy of follow-up of cohorts. Detailed information of screening is shown in Table 2.

**Table 2:** Quality assessment for studies included

Year of publication	Authors	Selection				Comparability	Outcome			Total
		Representative participants	Illustration of non-exposed cohort	Ascertainment for exposure	No endpoints presented at beginning		Definition of endpoints	Long enough time for exposure	Adequacy of follow-up	
2019	Keyong Huang et al.	**✵**		**✵**	**✵**	**✵✵**	**✵**	**✵**	**✵**	8
2018	Yutong Cai et al.	**✵**		**✵**	**✵**	**✵✵**	**✵**	**✵**		7
2018	GeorgeS Downward et al.	**✵**		**✵**	**✵**	**✵✵**	**✵**	**✵**		7
2018	Jong-Hun Kim et al.	**✵**	**✵**	**✵**		**✵✵**	**✵**	**✵**		7
2017	Hong Qiu et al.	**✵**	**✵**	**✵**		**✵✵**	**✵**	**✵**		7
2019	Saeha Shin et al.	**✵**		**✵**	**✵**	**✵✵**	**✵**	**✵**		7
2019	Juhwan Noh et al.	**✵**		**✵**	**✵**	**✵✵**	**✵**	**✵**		7

#### Bias from characteristics of population

Cohorts of the 7 studies selected average located residents for studies’ participants which covered people in urban and rural regions, men and women, adults of all ages, and other aspects; thereinto, 3 studies (Downward et al. 2018; Shin et al. 2019; Noh et al. 2019) included the occurrence of immigration or the inhabitants’ movement in the research areas. One study (Kim et al. 2018) did not report the detailed information or characteristics of research population, and one study (Qiu et al. 2017) selected the elder whose age was over 60 years; moreover, these 2 studies were solely scored in reports of the non-exposed cohort.

#### Bias from exposure measurement

Data for ambient PM_2.5_ was acquired through satellite-based model in 5 studies, and data was collected from outdoor-automated monitoring stations in other 2 studies (Noh et al. 2019; Downward et al. 2018). With regard to extent of ambient PM_2.5_ increasing, the least level (Cai et al. 2018) was 1.4 μg/m^3^ and the highest one (Huang et al. 2019; Qiu et al. 2017; Kim et al. 2018; Noh et al. 2019) was 10 μg/m^3^. The range of exposure period was 3 (Cai et al. 2018) to 20 years (Qiu et al. 2017). The earliest observation (Kim et al. 2018) began in 1990, and the last cohort study (Huang et al. 2019; Shin et al. 2019) began in 2015.

#### Bias from covariate adjustment

Data processing for all 7 cohort studies was performed via proportional hazards models adjusted by different covariates. Mutual covariates were age (4 studies), gender (5 studies), education (4 studies), body mass index (4 studies), and smoking status (5 studies). Besides, 2 studies exhibited crude HR (Cai et al. 2018; Noh et al. 2019) and the data in a sole study (Kim et al. 2018) did not adjust any covariate.

#### Bias from definition of endpoint

All endpoints of included studies were defined according to items of International Classification of Diseases (ICD). Nevertheless, different editions and ranges of items in detail might result in bias of the meta-analysis. Three studies (Kim et al. 2018; Noh et al. 2019; Huang et al. 2019) only adopted content in ICD-10. Among them, one study (Kim et al. 2018) reported the most detailed items. Both ICD-9 and ICD-10 were used in 2 studies (Cai et al. 2018; Shin et al. 2019). Yet one study (Downward et al. 2018) did not mention editions or items of ICD and the last one (Qiu et al. 2017) only used ICD-9.

### Increase of PM_2.5_ and HS

#### Total outcome

**Fig. 2:**
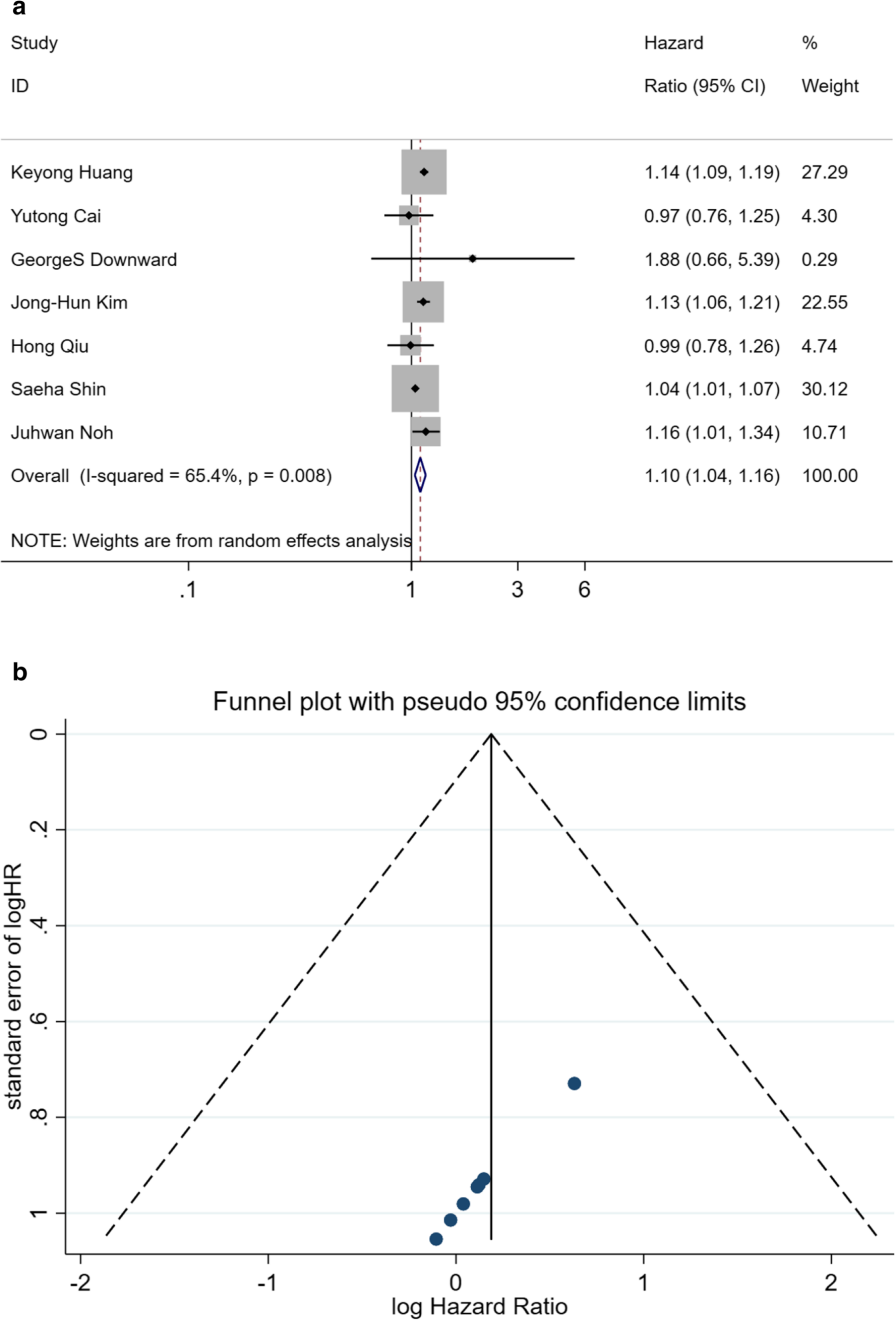
**a** Pooled HR for crude HRs. **b** Funnel diagram and Egger’s regression of crude HRs

The crude HRs or HRs adjusted for the least factors of all 7 studies were shown in Fig. 2a, including exposure to PM_2.5_ increase and incidence or mortality of HS. The pooled HR for each 1.4–10 μg/m^3^ increase in PM_2.5_ was 1.10 (95%CI: 1.04–1.16), which indicated a positive association between exposure to growing PM_2.5_ and incidence or mortality of HS. Yet the heterogeneity of these studies was significant (I^2^ = 65.4%, *p* = 0.008). There was no publication bias according to Funnel plot and Egger’s regression (*p* = 0.225, Fig. 2b). Therefore, to attenuate the heterogeneity, sensitivity analysis was performed. Crude HRs were omitted one by one, and pooled HRs of the rest studies were calculated. All the pooled HRs, 95%CI, I^2^, and *p* are shown in Table 3. The difference between I^2^ was readily discernible. The heterogeneity did not disappear (*p* = 0.599) until the study (Shin et al. 2019) would be deleted. This might be the HR of the study was from a proportional hazards models adjusted by the least covariates. After a comprehensive survey of covariates in other studies, recent immigrants, income quintile, urban/rural area, and northern/southern Ontario might be special covariates that led to the bias. As the study was omitted, the pooled HR was 1.13 (95%CI: 1.09–1.17).

**Table 3:** Crude HRs omitted one by one and pooled HRs of the rest studies

Article deleted	Pooled HR (95%CI)	Test of heterogeneity	
		I^2^ (%)	*p*
Keyong Huang et al.	1.08 (1.02, 1.14)	42.0	0.125
Yutong Cai et al.	1.10 (1.04, 1.17)	70.1	0.005
GeorgeS Downward et al.	1.09 (1.03, 1.16)	69.2	0.006
Jong-Hun Kim et al.	1.09 (1.01, 1.16)	66.7	0.010
Hong Qiu et al.	1.10 (1.04,1.17)	70.3	0.005
Saeha Shin et al.	1.13 (1.09,1.17)	0.0	0.599
Juhwan Noh et al.	1.09 (1.02,1.16)	69.2	0.006

#### Subgroup analysis

**Fig. 3:**
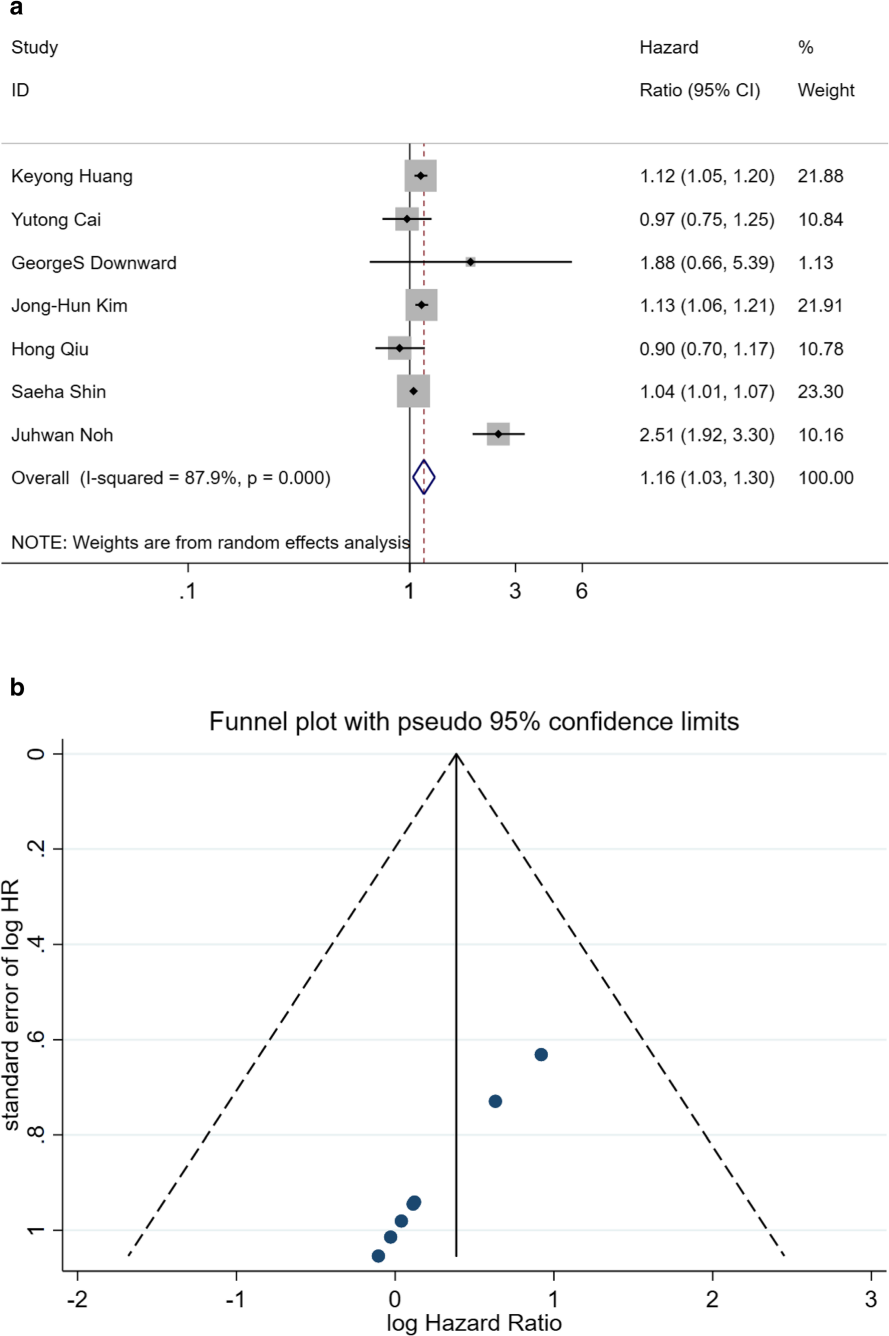
**a** Pooled HR for HRs adjusted for different covariates. **b** Funnel diagram and Egger’s regression of HRs adjusted for covariates

Adjusted HRs for the most factors of all 7 enrolled studies are shown in Fig. 3a. The pooled HR for each study was 1.16 (95%CI: 1.03–1.30), which indicated a positive association between exposure to growing PM_2.5_ with different covariates and incidence or mortality of HS. Yet the heterogeneity of these studies was significant (I^2^ = 87.9%, *p* = 0.000). Publication bias was not evident according to Funnel plot and Egger’s regression (*p* = 0.401, Fig.. 3b). Therefore, sensitivity analysis was conducted to attenuate the heterogeneity. Adjusted HRs were omitted one by one, and pooled HRs of the rest studies were re-calculated. All the pooled HRs, 95%CI, I^2^, and *p* are shown in Table 4; however, the I^2^ was not attenuated.

**Table 4:** HRs adjusted for covariates omitted one by one and Pooled HRs of the rest studies

Article deleted	Pooled HR (95%CI)	Test of heterogeneity	
		I^2^ (%)	*p*
Keyong Huang et al.	1.19 (1.01, 1.39)	89.4	0.000
Yutong Cai et al.	1.18 (1.05, 1.34)	89.8	0.000
GeorgeS Downward et al.	1.15 (1.03, 1.29)	89.7	0.000
Jong-Hun Kim et al.	1.18 (1.01, 1.38)	89.2	0.000
Hong Qiu et al.	1.19 (1.06,1.35)	89.5	0.000
Saeha Shin et al.	1.22 (1.02,1.44)	87.0	0.000
Juhwan Noh et al.	1.08 (1.02,1.14)	54.6	0.051

**Fig. 4:**
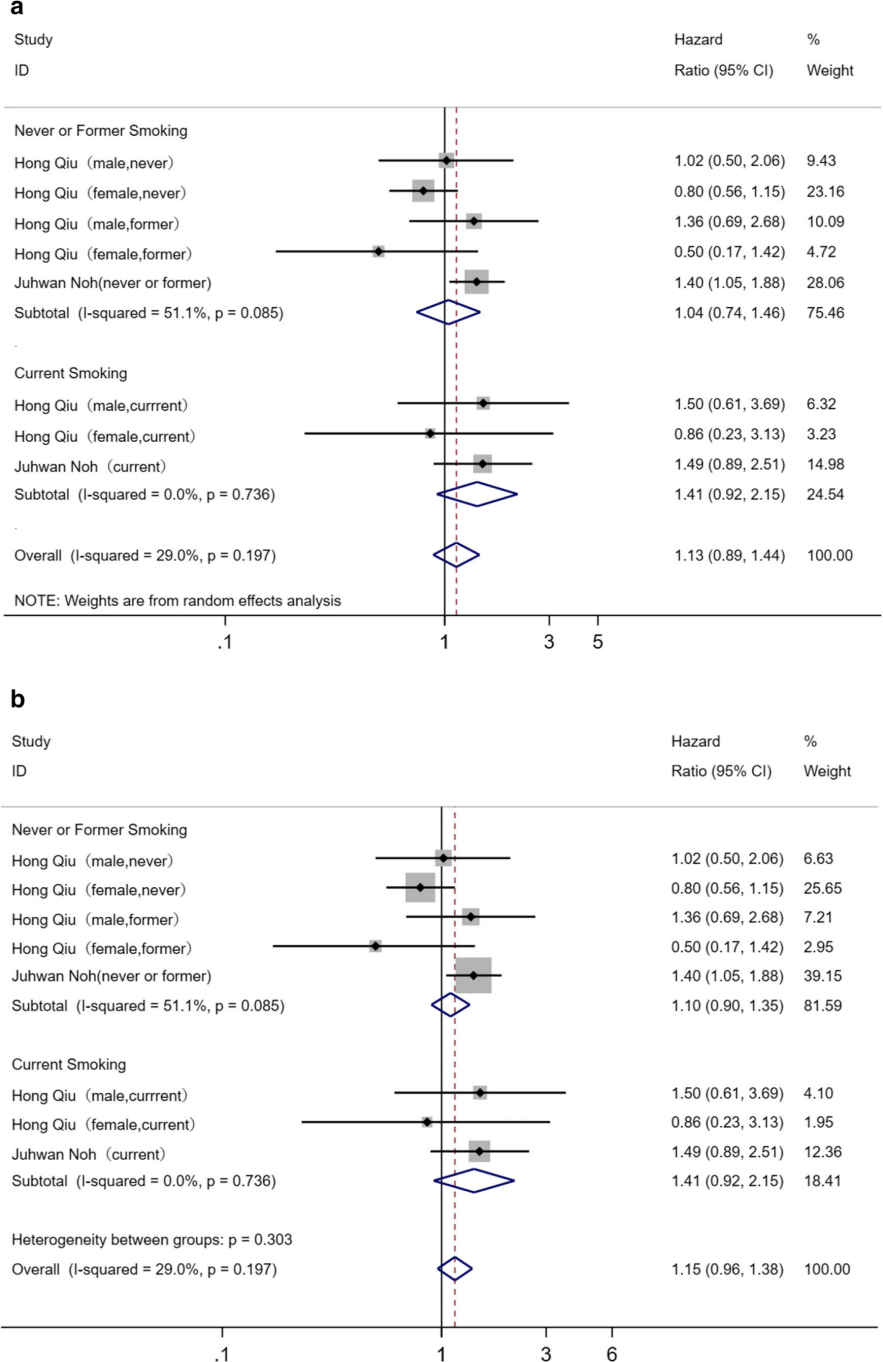
**a** Subgroup analysis for smoking. **b** Sensitivity analysis for subgroup analysis by switching effects models

According to the definite fact that smoking could damage the vascular endothelium, smoking might aggravate the effect of PM_2.5_ increment to brain vessels. Thus, subgroup analysis was performed. For individuals who never smoke and those who were former smokers versus individuals who were currently smoking, significant association between exposure to growing PM_2.5_ and incidence or mortality of HS was found (never or former smoking, pooled HR = 1.04, 95%CI: 0.74–1.46; current smoking, HR = 1.41, 95%CI: 0.92–2.15). Subgroup analysis and its sensitivity analysis carried out through switching the random effects model to fixed effects model is shown in Fig. 4 a and b.

## Discussion

PM_2.5_ is a kind of the inhaled particulates that could enter the circulatory system via permeating alveolar epithelium and vascular endothelium. Therefore, PM_2.5_ might be transmitted to the arteries in the brain and deposited on the surface of vascular endothelium, further leading to HS. Thus, effect of deposition or particulates trundle might result in inflammation and injury of vascular endothelium. Finally, the suffered arteries might have corrosion damaging the vessel wall.

Chronic diseases (To et al. 2015) including angina, asthma, congestive heart failure, and diabetes are closely related to PM_2.5_. Many articles reported relationship between long-term exposure to PM_2.5_ and increased risks of incident stroke (Atkinson et al. 2013; Lipsett et al. 2011; Ljungman et al. 2019; Lin et al. 2016; Korek et al. 2015; Hoffmann et al. 2015; Dirgawati et al. 2019) and ischemic heart disease (Carey et al. 2016; Hartiala et al. 2016; Katsoulis et al. 2014; Loop et al. 2018; Ueda et al. 2012; Villeneuve et al. 2015; Stockfelt et al. 2017). A case-control study (Qian et al. 2019) reported that fatal intracranial hemorrhage incidence was associated with PM_2.5_ exposure and diabetes might increase the risk for intracerebral hemorrhage incidence in relation to PM_2.5_. In this meta-analysis, we found the evidence that exposure to different levels of ambient PM_2.5_ per unit was related to risk of HS in cohort studies. Sheng Yuan et al. (Yuan et al. 2019) concluded that pooled HR for stroke and long-term exposure to 5 μg/m^3^ increment of PM_2.5_ was 1.20 (95%CI: 0.79–1.80; I^2^ = 64.2%, *p* = 0.039) and the subgroup analysis outcomes of smoking status were 1.08 (95%CI: 1.03–1.13; I^2^ = 12.3%, *p* = 0.334) for never smoking, 1.11 (95%CI: 1.01–1.22; I^2^ = 0, *p* = 0.898) for former smoking, and 1.08 (95%CI: 0.94–1.25; I^2^ = 0, *p* = 0.462) for current smoking. This meta-analysis included less literatures than previous studies and had un-unified PM_2.5_ increment levels. Besides, due to limited data of included studies, the subgroups in this meta-analysis were divided into the current smoking subgroup and the subgroup of never smoking and former smoking.

In perspective of recommendations for further research about relationship between PM_2.5_ and risk of HS, a prospective cohort study is the first choice of study design selection. In the environment exposed by participants, the different levels of PM_2.5_ should preferably be obvious and balanced. All participants do not present the endpoint (such as HS) at the beginning period of a study. Exposure measurement is related to the sensitivity and accuracy of the outcome; therefore, the third scientific institutions and proper mathematical models would be used. As to adjustment for covariates, according to this meta-analysis, educated levels, classification of body mass index, and different age grade might be new researchable variables, which can be arranged to appropriate scales for subgroup analysis in the future. Definition of endpoints would be standardized and detailed according to professional items in the world; especially, adequacy of follow-up of cohorts will be manifested in articles.

As previously mentioned, there were several limitations of this systemic review. Only 7 articles from 3 open electronic databases during 2017 to 2019 were included in meta-analysis. Therefore, those not published in open platform were lost. Moreover, increment of PM_2.5_ was not unified, which ranged from 1.4 μg/m^3^ to 10 μg/m^3^. Numerous covariates were concealed in data synthesis, which might result in bias or heterogeneity of the meta-analysis and subgroup analysis.

## Conclusion

Ambient PM_2.5_ level was significantly associated with the risk of HS, which might be a potential risk factor of HS. Additionally, under exposure of PM_2.5_, smoking does not further increase the risk of HS.

## Data Availability

All the data and material pertinent to this manuscript are included and have been reviewed by all authors.

## Appendix

The crude data extracted from literature included.
